# A systematic review and meta-analysis in the effectiveness of mobile phone interventions used to improve adherence to antiretroviral therapy in HIV infection

**DOI:** 10.1186/s12889-019-6899-6

**Published:** 2019-07-09

**Authors:** Reshma Shah, Julie Watson, Caroline Free

**Affiliations:** London School of Hygiene and Tropical Medicine (LSHTM), London, UK

**Keywords:** HIV, Technology, Medication adherence, Mobile phone, Health behaviour

## Abstract

**Background:**

Antiretroviral therapy is effective in preventing the progression of HIV to AIDS, but adherence to HIV medication is lower than ideal. A previous Cochrane review concluded that SMS interventions increased adherence to HIV medication, but more recent trials have reported mixed results. Our review aims to provide an up-to-date synthesis of the effects of interventions delivered by mobile phone on adherence.

**Methods:**

We searched Cochrane, Medline, CINAHL, EMBASE and Global Health for randomised control trials (RCTs) of interventions delivered by mobile phones, designed to increase adherence to antiretroviral medication. Risk of bias was assessed using the Cochrane risk of bias tool. We calculated relative risk ratios (RR) or standardised mean difference (SMD) with 95% confidence interval (CI). Trials were analysed depending on delivery mechanism and intervention characteristics. We conducted meta-analysis for primary objective outcome measures.

**Results:**

We identified 19 trials. No trials were at low risk of bias. Interventions were delivered as follows; nine via text message, five via mobile phone call, one via mobile phone imagery and four via mixed interventions. There was no effect when interventions delivered by text message were pooled in the RR1.25 (CI 0.97 to 1.61) *P* = 0.08. The SMD 0.42 (0.03 to 0.81) *p* = 0.04 showed a moderate effect to improve adherence. There was mixed evidence of the effect of text messages delivered daily, weekly, at scheduled or triggered times, however, messages with link to support, interactivity and three or more behavior change techniques (BCTs) all improved adherence. Of the five trials delivered by mobile phone call, one reported a reduction in HIV viral load. One trial using mobile phone imagery reported a reduction in HIV viral load. Three trials that delivered interventions by text message and mobile phone counselling reported improved biological outcomes.

**Conclusion:**

Specific interventions, of proven effectiveness should be considered for implementation, rather than mobile phone-based interventions in general. Interventions targeting a wider range of barriers to adherence may be more effective than existing interventions. The effects and cost-effectiveness of such interventions should be evaluated in a randomised controlled trial alongside long term objective and clinically important outcomes.

**Electronic supplementary material:**

The online version of this article (10.1186/s12889-019-6899-6) contains supplementary material, which is available to authorized users.

## Background

There are currently over 36 million people worldwide living with HIV [1] with the majority from middle and low-income countries. Almost 70% of the global HIV disease burden is in Sub-Saharan Africa [2]. Treatment with antiretroviral therapy (ART) enables people living with HIV (PLWH) to lead healthier and longer lives since the life expectancy of someone who responds to treatment is the same as the general population [3]. Currently, 59% of PLWH have access to ART [1].

High adherence to ART is required to suppress viral replication, to slow the progression of HIV, and further reduce transmission [4]. Poor adherence can also lead to drug resistance [5]. UNAIDS aims to ensure 73% of PLWH achieve viral suppression which is thought to be 2 to 3 times higher than current levels of viral suppression [6] and the World Health Organisation (WHO) estimates that only one-third of the population adhere appropriately [7].

Factors influencing adherence include individual factors such as lack of knowledge, misunderstandings about administering medicines, lack of skills in developing regular medicine taking habits (remembering), concerns about side effects, and social support for medicine taking [8–10]. Medicine related factors, which affect adherence, include pill burden (number of tablets, intense dosing schedule, meal time restrictions, medicine side effects) [11–13]. Service and structural factors also play a role, such as the availability of medicine and cost of training health care providers [14, 15].

Interventions that have demonstrated efficacy in increasing adherence to HIV treatment have had multiple components including providing education, counselling, social support, feedback and additional supervision [11–13]. However, in most settings these have proven too costly or are unfeasible to deliver in routine service settings.

Mobile phones are a potentially useful, low cost, platform for delivering health interventions [16]. The World Bank estimates that 93 in every 100 people are subscribed to mobile phones [17] with low-income countries being the fastest growing sector [18]. Interventions delivered by mobile phones have the potential to target many of the factors influencing adherence such as knowledge, attitudes, concerns about medicines and difficulties in developing regular medicine taking habits [8–10]. They could enhance links to services so participants can obtain support and advice when needed, such as if they are experiencing medicine side effects [19]. Where mobile phones are owned and used by individuals, privacy can be maintained which is vital for stigmatised diseases like HIV. Mobile phones are carried with people wherever they go, so advice and support can be provided in real time in the patient’s environment [20]. Mobile phones also have the potential to provide support and training to health care providers, allow remote monitoring of medicine taking and monitoring of drug supplies with the potential to reduce drug stock.

A 2011 Cochrane systematic review of trials conducted between 1980 and 2011, included two trials of interventions delivered by mobile phone and concluded weekly messaging is effective in increasing adherence to ART [19]. Other systematic reviews that have looked at text message also support these findings; Finnitis et al. [21], Mayer et al. [22] and Thakker et al. [23] suggest text messaging improves adherence. Wald et al. [24] looked at the difference between one-way and two-way message and found the latter improved adherence.

Our review aims to provide an updated synthesis of RCTs of interventions designed to increase adherence to ART medication delivered to patients via mobile phone. We aim to describe the effectiveness of interventions which employ different delivery mechanism (SMS, voice calls, application software) and different intervention content or frequencies of contact (weekly, daily contact). We will explore if the effects of interventions vary according to if they employ interactivity, links to support or use three or more BCTs.

## Methods

This review was conducted in accordance with the PRISMA guidance. The flowchart can be found in Additional file 1.

### Inclusion criteria

Participants – Men and women of any age infected with HIV who are on or due to start ART. There was no restriction of age or stage of treatment.

Intervention – All controlled trials employing any mobile technology to deliver interventions to improve adherence to antiretroviral medication.

Study design – Randomised control trials.

Outcomes – Primary outcomes were objective measures, which include Medication Event Monitoring System (MEMS), pill count and biological outcomes (CD4 count and viral load). Secondary outcomes were subjective measures (self-reported adherence).

A review protocol does not exist. There were no language, geographical or publication status restrictions. We excluded trials that included more than one disease and all non-randomised trials including observational and cross-sectional study designs.

### Search strategy

We searched; Cochrane, CINAHL, MEDLINE, EMBASE, and Global Health databases from 1990 to October 2017. The EMBASE search strategy of medical subject headings and text-words can be found in Additional file 2. These terms were combined with the Cochrane pre-set search terms for controlled trials. RS searched the reference lists of included papers to identify additional studies for this review. Two reviewers independently scanned the electronic records to identify potentially eligible trials.

### Data extraction

Two reviewers independently extracted data on the intervention delivery mechanism (e.g. text message, phone call), intervention characteristics, trial quality and on measures of effect. Sensitivity analysis was run looking at intervention characteristics, defined as:

(i) Link to support - an intervention that was linked to a health professional was considered as support, e.g. the provision of a telephone number.
(ii) Interactivity - when an intervention required the participant to respond once the intervention has been received for e.g. sending a text message back that “everything is okay”. Also referred to as two-way text message.
(iii) Behaviour change technique - authors description of interventions according to Abraham and Michie’s taxonomy of BCTs [25], Additional file 3. An arbitrary measure of three or more techniques were used as a cut-off as we estimate that this number is indicative of interventions which have considered behaviour change and a wider range of factors influencing adherence.

All discrepancies were agreed by discussion with a third reviewer.

Risk of bias was assessed according to the criteria outlined by the International Cochrane Collaboration [26]. A cut off of 90% complete follow-up was used to determine low risk of bias for attrition.

### Data analysis and synthesis

All analyses, including meta-analysis were conducted in Cochrane Review Manager [27]. All outcomes have been analysed as intention-to-treat. All loss to follow up has been treated as non-adherent. We calculated risk ratios (RR) and standard mean differences (SMD). We used random effects meta-analysis appropriately to give pooled estimates of primary outcomes where there were two or more trials using the same mobile phone delivery mechanism (e.g. SMS messages) and the same measures of adherence. We examined heterogeneity visually by examining the forest plots and statistically using both the χ^2^ test and the I2 statistic. We assessed evidence of publication bias using Funnel plots.

## Results

The combined search strategies identified 511 electronic records. These were screened for eligibility and the full text of 46 potentially eligible reports were obtained for further assessment. Nineteen reports met the review inclusion criteria and represented 19 trials, the PRISMA flow diagram can be seen in Additional file 1. Trials that were excluded from this review can be found in Additional file 7. Three trials were excluded in meta-analysis; Nsagha [28] and Kebaya [29] only reported subjective outcomes and Hardy [30] had an intervention as part of the control group.

### Trial characteristics

In total, the 19 trials included 2650 participants. Sample sizes ranged from 21 to 538 people, the median was 119 participants. Studies took place in South America (Brazil = 1), North America (USA = 4), Asia (India = 1, Pakistan =1, Malaysia =1, China = 2), Africa (Nigeria =1, Cameroon = 2, Uganda =1, South Africa = 1, Kenya = 3) and Australasia (New Zealand =1). All were presented in English. Most trials recruited men and women, however, two groups included only women [29, 31].

### Interventions – delivery mechanism

Nine interventions were delivered via text message [28, 30–37], five interventions were delivered via mobile phone call [29, 38–41], one via mobile phone imagery [42], and four mixed interventions (one automated voice call with pictorial text [43]), two with counselling and text message [44, 45] and one with SMS and telephone call reminder [46]. In all but one trial the control was usual care [30]. The interventions lasted between 6 weeks and 96 weeks.

The maximum number of BCTs employed was 6 [41] and the median number was 2. The most commonly used BCTs were: social support (13 interventions), association, for example prompts/cues (11 interventions) and feedback & monitoring (8 interventions). More than three BCTs were used in 5 of the trials [34, 35, 38, 40, 41]. Four trials specifically reported being based on behaviour change theory [33, 35, 41, 43] and these include social cognitive theory of planned behaviour [43, 47], health belief model [33], individual change model [35], and behavioural self-management [41].

Of the 19 mobile phone interventions, five were interactive [30, 35, 43–45]. A link to support was provided in six trials [33, 38, 39, 41, 44, 45].

Details of the interventions, as described by the authors are described in Table 1.

**Table 1:** Details of included studies

Trial	Delivery mechanism	Trial design, Country, Device, Media, Intervention length	Participants	Aims	Intervention	Behavioural Change Theory, interactivity, link to support	Abraham and Michie Taxonomy	Comparator
**TEXT MESSAGE**								
Da Costa 2012 [31]	Text message 5 times a week	RCT, Brazil, mobile phone, SMS, 4 month	21 HIV infected adults. 100% female	To assess whether a warning system based on mobile SMS increases the adherence (95%) of HIV-infected Brazilian women to ART regimens and their impression and satisfaction with respect to incoming messages.	Participants received SMS messages 30 min before their last scheduled time for a dose of medicine during the day. Sent Saturday, Sunday, and alternate days during the working week		Social support 3.1, prompts 7.1	Monthly multidisciplinary attendance, no text messages
Pop-Eleches 2011 [32]	Weekly, daily, short and long text message	Parallel group RCT 5 arms, Kenya, mobile phone, SMS, 48 weeks	431 HIV infected adults, initiated ART less than 3 months ago. 66% female.	To test the efficacy of SMS reminders on adherence (90%) to ART among patients attending a rural clinic in Kenya.	Participants received SMS reminder that were either short or long and sent at a daily or weekly frequency.		Social support 3.1, prompts 7.1	No SMS messages
Mbuagbaw 2012 [33]	Weekly text message	Parallel RCT, Cameroon, mobile phone, text message, 6 months	200 HIV positive adults, 21 years and above, been on ART for 1 month. 73.5% female.	To test the effectiveness of sending weekly motivational text messages via mobile phone to improve adherence.	Weekly standardised motivational text message	Health belief model of behaviour change. Link to support - SMS contained a phone number they could call back if they needed help	Social support 3.1, prompts 7.1	Standard care and no text message
Hardy 2011 [30]	Daily text message	Parallel RCT, America (Boston), mobile phone, message, 6 weeks	23 HIV infected adults, on ART for at least 3 months but less than 85% adherence. 39% female.	To compare the efficacy of a personalized cell phone reminder system (A remind) in enhancing adherence to ART versus a beeper.	Personalised text messages daily to match ART dosing frequency, beep every 15 min till patient acknowledges message.	Interactivity - participant instructed to respond with an SMS in order to confirm they were taking medication. It would bleep every 15 min	prompts 7.1	Beeper, reminder beeper at the time of dosing, beeps once for 30 s then no repeats.
Sabin 2015 [34]	SMS text reminder	RCT, China, 9 month	119 participants. Age 18+, deemed at risk of poor adherence, 36.1% female	Hypothesized that adherence information and education likely to be effective when delivered in real time and in direct response to lapses when they occur. To assess the effect of real-time feedback using triggered cell phone reminders coupled with Wisepill generated data enhanced counselling.	Adherence counselling and SMS phone reminder when the Wisepill system failed to detect 30 mins post scheduled dose time. Monthly clinic if adherence < 95% - received behaviourally targeted counselling session with a counsellor guided by performance report		Feedback on behaviour 2.2 Prompt 7.1 Social support 3.1	Monthly clinic if adherence < 95% - received behaviourally targeted counselling session with a counsellor guided by performance report
Ingersoll 2015 [35]	Automated SMS text message	RCT, USA (Virginia), 12 weeks	63 participants. Age 18+, 39.7% female. Less than 95% adherence used illicit drugs and drank risky amounts of alcohol	Test the preliminary efficacy of a theory based bi-directional text message intervention on ART adherence, missed care visits and substance use among people with HIV	Daily queries of ART adherence, mood and substance use. The system sent contingent intervention messages by participants for reports of medication dosing, mood and substance use	Interactivity – bidirectional texting Behaviour change Theory – Information, motivation and behaviour skills model of adherence and social action theory. Individual change model	Social support 3.1, Monitoring of emotional consequences 5.4, Prompt 7.1	HIV primary care, speciality service, medical case management, pharmacist adherence support, psychological care and substance abuse counselling
Haberer 2016 [36]	SMS message	RCT, Uganda, 9 months	62 people with HIV, 65% female and their social supporters.	RCT of multiple types of interventions based on SMS and real time adherence monitoring to improve adherence among individuals initiating ART in Uganda	1) Scheduled SMS – SMS daily for 1 month, then weekly for 2 months, then for the next 6 months only if no signal within 2 h, and SMS to social supporter if no signal 48 h + real time adherence monitoring 2) Triggered SMS only if no signal within 2 h of dosing, last 6 months SMS sent to social supporter + real time adherence 3) real time adherence only		Feedback on behaviour 2.2, Social support 3.1, Prompt 7.1 Social supporter had a text message	Real time adherence no SMS reminders
Nsagha 2016 [28]	SMS message	RCT, Cameroon, 1 month	90 people living with HIV and AIDS who had been on ARV for 1 month. 61% female, aged 18 and above 95% adherence	To assess the usefulness of cell phone text messages to improve the adherence (95%) of HIV and AIDS patients to their treatment and care in the NW region of Cameroon	Educative SMS messages, four times a week for 4 weeks		Prompt 7.1	Usual care – no details
Orrell 2015 [37]	Text message	RCT South Africa, 48 weeks	230 ART naïve. 65.2% female. Adherence more than 80%	Determine if text message triggered by missed doses would improve overall daily adherence execution in ART naïve South African adults	Participants preferred daily dosing time recorded in the EMS and if device not opened within 30 min of scheduled dosing time – text message sent.		Feedback on behaviour 2.2 Prompt 7.1	3 group treatment preparedness sessions before or within the first month of commencing ART. Delivered by HIV positive peer counsellors.
**VOICE CALL**								
Belzer 2014 [38]	Daily phone call	Longitudinal RCT, America, mobile phone, voice calls, 48 weeks	37 youth (age 15-24) with HIV, history of non-adherence. 37% were females. Half the youth were perinatally infected.	To determine if daily cell-phone conversation with health-care providers around self-care and taking HIV management would lead to successful self-administration of ART in HIV infected adolescents with poor medication adherence (< 90%).	Calls Monday to Friday either once or twice a day. Confirmed if medications were taken, provided problem-solving support and referred to services to address adherence barriers.	Link to support - adherence facilitator did a mediation review, problem solving and scheduling relevant referrals.	Goals and planning 1.2, 1.6, Feedback and monitoring 2.2, Social support 3.1, prompts 7.1	Usual care
Huang 2013 [39]	Fortnightly phone call	RCT, China, mobile phone, voice call, 3 months	196 HIV positive patients, both treatment naïve and treatment experienced. 52% female.	To investigate the effects of a phone call intervention on adherence to ART and QOL of treatment-naïve and treatment-experienced patients.	Reminder phones call every 2 weeks by registered nurse/ health personnel. Discuss medications, self-management and related difficulties.	Link to support - patients were given a hospital phone number and a mobile phone number	Feedback and monitoring 2.1, Social support 3.2	Usual care - educational on HIV/AIDS and treatment
Kebaya 2014 [29]	Fortnightly mobile phone call	RCT, Kenya, mobile phone, voice call, 6 weeks	150 mother- infant pairs, in HIV exposed infants.	To compare self-reported adherence to infant NVP prophylaxis and retention in care in.	Fortnightly mobile phone based reminder and on prevention of mother to child transmission.		prompts 7.1	Standard health care, no phone calls.
Uzma 2011 [40]	Weekly phone call reminders	RCT, Pakistan, mobile phone, voice call, 10 weeks	76 adult participants, 26.3% females, HIV positive on ART regime for at least 3 months	To assess the efficacy of interventions for improving adherence to ART regimens in patients with HIV/AIDS in treatment centres.	Weekly phone reminders and routine counselling		Goals and planning 1.4, Social support 3.1, prompts 7.1	Routine counselling
Kalichman 2011 [41]	Bi-weekly phone call counselling session	RCT, America (Atlanta), mobile phone, telephone counselling session, 4 months	40 HIV positive adults with less than 95% self-reported adherence. 35% female.	To examine the effect of a brief cell phone-delivered adherence intervention designed to improve medication adherence in people living with HIV/AIDS	45 min counselling session with feedback and adherence counselling after pill count calls, which were bi-weekly.	Behavioural self management model, self-regulation models of medication adherence Link to support - counsellor initiated calls and check in on how participants were doing	Goals and planning 1.1, Feedback and monitoring 2.2, 2.3, Social support 3.1, 3.2, Natural consequence 5.1	Pill counts checked over the phone, no feedback on adherence or counselling.
**MIXED**								
Maduka 2013 [45]	Mixed- twice a week text message and monthly adherence counselling	RCT, Nigeria, mobile phone, short message reminders, 4 months	104 participants, 56.7% female, HIV positive been on HAART for 3 months, history of non-adherence	To demonstrate the effect of adherence counselling and text message reminders in improving patient’s adherence to HAART.	Mixed - monthly adherence counselling that lasted 45 - 60 min, and twice weekly short message reminders	Interactivity - trial participants were encouraged to call, “flash” or send an SMS to those numbers to acknowledge receipt of SMS and indicate need for further counselling or information Link to support - researchers phone contact were added to the receiver category of every message	Social support 3.1, prompts 7.1	Standard care -included health education, occasional encouragement by doctors & quarterly assessment of CD4 count.
Lester 2010 [44]	Weekly text message + counselling if required	Parallel group RCT, Kenya, mobile phone, SMS, 12 months	538 HIV infected, ART naïve adults. 65.6% female.	Assess whether mobile phone communication between health-care workers and patients initiating ART, in Kenya improved drug adherence and suppression of plasma HIV-1 RNA load.	Weekly SMS messages that required a response within 48 h. Structured mobile phone communication between health care workers and patients.	Interactivity - instructed to respond after 48 h if they were doing well or if they had a problem. Those that had a problem the clinician called them back or those who failed to respond. Link to support - SMS reminded them of phone based support	Social support 3.1	One counselling sessions in Kajido and two in Nairobi. Additional brief counselling at each site provided during dispensation of the drugs in the clinic or pharmacy.
Shet 2014 [43]	Automated weekly voice message + weekly pictorial message	Parallel group RCT, India, mobile phone, automated voice call and pictorial message 96 weeks	631 HIV infected, ART naïve adults. 43.3% women.	To assess whether customised mobile phone reminders would improve adherence to therapy and thus decrease virological failure among HIV infected patients started ART	Customised, interactive automated voice reminder and a pictorial message sent weekly to the patient’s mobile phones.	Social cognitive theory of planned behaviour Interactivity - required patient to respond about previous days dosing. If there was no response to the call then three more attempts were made until a response was obtained.	Feedback and monitoring 2.1, Social support 3.1	Standard care included 3 counselling sessions prior to the initiation of ART, routine clinical and lab testing at baseline, follow up and assessments every 6 months
Abdulrahman 2017 [46]	SMS and telephone call reminder	Single blinded RCT, Malaysia, SMS and telephone call reminder + adherence counselling, 24 weeks	242 Adult HIV positive new to ART. 12% female. Excluded pregnant/HIV patients already on/restarted ART and foreigners.	Evaluate the effectiveness of mobile phone reminders and peer counselling in improving adherence and treatment outcome among HIV positive patients on ART in Malaysia	Weekly medication reminder SMS 3 days prior to app, telephone call reminders (90 s during lunch hours) for scheduled clinic appointments and peer counselling during clinical visits (minimum of 3 visits)	Link to support - not mandatory to respond but could text back for additional support	Prompt 7.1	Standard care – routine adherence counselling and paper based appointment scheduling by two training research assistants
**IMAGING**								
Perera 2014 [42]	Daily imaging	RCT, New Zealand, smartphone application, imagery, 3 months	28 adults on ART for at least 6 months, 7% female.	To examine the efficacy of a smartphone application incorporating personalized health related visual imagery with real-time information about medication level and immune-protection to enhance adherence to ART.	Augmented version contained components that illustrated participant’s current estimated plasma concentration of ART and the immune protection.		Feedback and monitoring 2.2, prompts 7.1	Standard version of smartphone application, which comprised a 24-h medication clock, displaying the participants daily ART dosing schedule. Patients could record when they had taken their medication each day.

### Outcomes

Fifteen different adherence outcome measures were reported. Primary objective outcome measures reported include six trials recordings MEMs, seven viral measures (5 reported viral load and 2 viral failure) and three trials measured pill count and CD4 count. The most frequently reported measure of adherence was the subjective secondary outcome, self-reported adherence, in 14 trials. A complete list is found in Additional file 4.

### Trial quality

A risk of bias summary for each trial is presented in Table 2 with comments in Additional file 5 [48]. No trials had a low risk of bias for all criteria assessed. A funnel plot to show publication bias can be found in Additional file 6.

**Table 2:** Summary risk of bias for each trial

Trial	Randomisation	Allocation concealment	Blinding – primary outcomes	Blinding – secondary outcomes	Incomplete outcome	Selective outcome reporting	Contamination	Other – general bias
Abdulrahman 2017	Low	Low	Low	High	Low	Low	Unclear	Unclear
Belzer 2014	Low	Unclear	Low	High	High	Low	Unclear	High
Da Costa 2012	Low	Unclear	Low	High	High	Low	Unclear	Unclear
Haberer 2016	Low	Unclear	Low	Low	Low	Low	Unclear	Unclear
Hardy 2011	Low	Unclear	Low	Low	High	Low	Unclear	Unclear
Huang 2013	Low	Low	Low	High	High	Unclear	Unclear	Unclear
Ingersoll 2015	Low	Low	Low	Low	Low	Low	Unclear	Low
Kalichman 2011	Low	Unclear	Low	Unclear	Low	Low	Unclear	Unclear
Kebaya 2014	Unclear	Unclear	Unclear	Unclear	Unclear	Unclear	Unclear	Unclear
Lester 2010	Low	Low	Low	High	High	Low	High	Unclear
Maduka 2013	Low	Low	Low	High	Low	Low	Low	Unclear
Mbuagbaw 2012	Low	Low	Low	High	High	Low	Unclear	Unclear
Nsagha 2016	Low	Unclear	Unclear	High	Unclear	Low	Unclear	Unclear
Orrell 2015	Low	Low	Low	Low	Low	High	Unclear	Unclear
Perera 2014	Unclear	Unclear	Low	High	Low	Unclear	Unclear	Unclear
Pop-Eleches 2011	Low	Unclear	Low	Low	Low	Unclear	Unclear	Unclear
Sabin 2015	Low	Low	Low	Low	Low	Low	Unclear	Unclear
Shet 2014	Low	Low	Low	Low	Low	Low	Low	Unclear
Uzma 2011	Low	Unclear	Low	High	High	High	Unclear	Unclear

### Interventions delivered by text message

Nine trials evaluated interventions delivered by text message [28, 30–37] which reported a total of 26 outcomes. An improvement in adherence was measured in seven of the 19 objective primary outcomes and two of the seven subjective secondary outcomes. The pooled measures of text message interventions to improve adherence measured as MEMs was RR 1.25 (CI 0.97 to 1.61) *P* = 0.08, observed heterogeneity I^2^ is 74% [31, 32, 34, 37] and SMD 0.42 (CI 0.03 TO 0.81) *P* = 0.04, I^2^ 28% [35, 36].

There was substantial variation in all the text message interventions. The frequency of text messaging was observed in Pop-eleches trial [32] which compared weekly and daily messages with the length of the message (long /short). Of these arms, only weekly text messages showed a significant result, which was not consistent in the Mbugbaw trial [33] where weekly text messages were also delivered. Three trials looked at daily text messages [30, 32, 35] of which two improved adherences [30, 35]. Some text message interventions were coordinated to their ART regime (scheduled) [30, 31] and others used real time monitoring which only sent a text reminder if the participant failed to open the medication device (triggered) [34, 36, 37]. Haberer [36] specifically looked at this function and split participants into scheduled and triggered. In this trial scheduled showed an effect, which was also supported by Hardy [30]. Triggered interventions showed an effect in two trials [34, 37]. Some trials only included participants with poor baseline adherence [30, 34, 35] and all of these trials reported that the intervention improved adherence. For biological measures, neither Orrell [37] or Haberer [36] reported a statistically significant HIV RNA suppression, however the Orrell trial [37] did report a statistically significant odds ratio for virological failure which has been asterisked in Table 3.

**Table 3:** summary tables of primary and secondary outcomes of interventions delivered by text message

Frequency of Interventions delivered by text message.	Trial	outcome	RR	SMD	LCI	UCI	*P* value
Primary outcome							
All text	Pop-eleches	Medication Events Monitoring	1.17		0.92	1.48	0.20
Weekly text	Pop-eleches	Medication Events Monitoring	**1.32**		**1.02**	**1.70**	**0.03**
Short text	Pop-eleches	Medication Events Monitoring	1.16		0.89	1.52	0.27
Long text	Pop-eleches	Medication Events Monitoring	1.17		0.90	1.53	0.24
Daily text	Pop-eleches	Medication Events Monitoring	1.01		0.76	1.35	0.92
Weekly text	Mbuagbaw	Pharmacy refill data		0.1	−0.23	0.43	0.62
Scheduled/5x week	Da Costa	Pill count	1.35		0.61	3.00	0.46
Scheduled/5x week	Da Costa	Mediation Events Monitoring	1.39		0.73	2.65	0.31
Daily text	Hardy	Medication Events Monitoring System		**33.4**	**14.1**	**52.6**	**0.00**
Scheduled/ Daily	Hardy	Pill count		13.7	−6.7	34.1	0.15
Scheduled/ Daily	Hardy	Composite adherence score		**27.1**	**7.6**	**46.6**	**0.01**
Triggered	Sabin	Medication events Monitoring	**1.69**		**1.28**	**2.20**	**0.00**
Scheduled	Haberer	Medication Events Monitoring		**12.00**	**1.83**	**22.17**	**0.03**
Triggered	Haberer	Medication Events Monitoring		0.00	−12.28	12.28	1.00
	Haberer	HIV RNA Suppression					0.14
Daily	Ingersoll	Pharmacy refill data		**12.20**	**1.11**	**23.29**	**0.04**
Triggered	Orrell	Medication Events Monitoring	1.02		0.90	1.15	0.73
Triggered	Orrell	Suppressed HIV RNA < 40 copies	1.05		0.93	1.18	0.46
Triggered	Orrell	Virological failure	*OR 2.03		**1.1**	**3.9**	**0.034**
Secondary outcome							
Weekly text	Mbuagbaw	Self-reported adherence	1.01		0.87	1.16	0.94
Weekly text	Mbuagbaw	Visual Analogue Scale > 95%	1.07		0.89	1.29	0.48
Weekly text	Mbuagbaw	Visual Analogue Scale > 90%	**1.14**		**1.01**	**1.30**	**0.03**
5 times a week	Da Costa	Self-reported adherence	1.06		0.83	1.35	0.65
Daily text message	Hardy	Self-reported adherence		20.2	−1.8	42.1	0.07
4 times a week	Nsagha	Self-reported adherence	**1.53**		**1.02**	**2.29**	**0.04**
Triggered	Orrell	Self report/ tablet return	1.00				1.00

Clinically significant results (*P* < 0.05) have been highlighted in bold

* refers to odds ratio

Interactivity was identified in 2 trials [30, 35] and both reported statistically significant improvements in adherence. Three or more BCTs were identified in 3 trials [34–36], all of which reported an improvement in adherence. Mbuagbaw [33] and Ingersoll [35] stated a behaviour change model that underpinned the intervention. Link to support was reported in one trial [33] which had a statistically significant effect when the adherence threshold was reduced from 95 to 90% for Visual Analogue Scale (VAS).

**Fig. 1:**
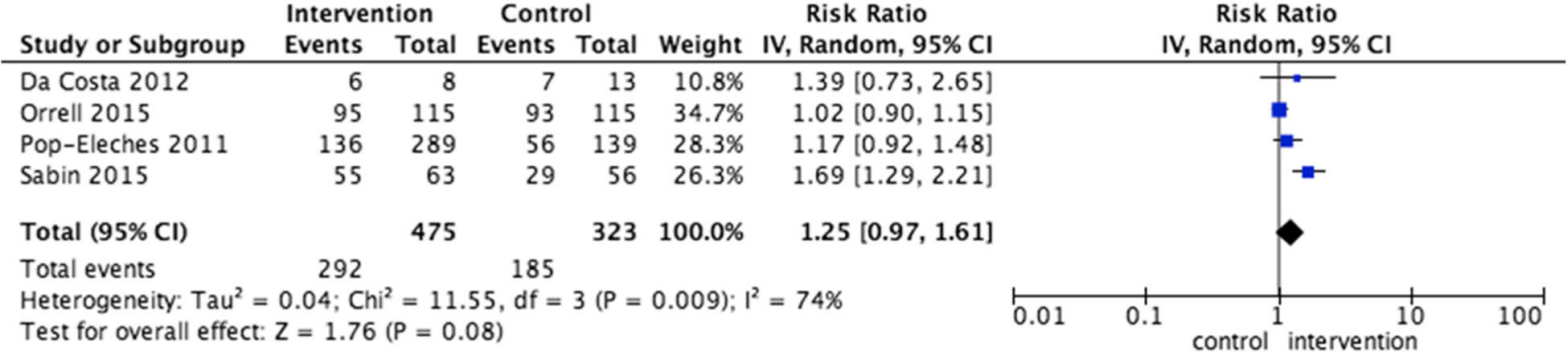
Adherence to HIV medication measured as MEM with interventions delivered by text message RR

**Fig. 2:**
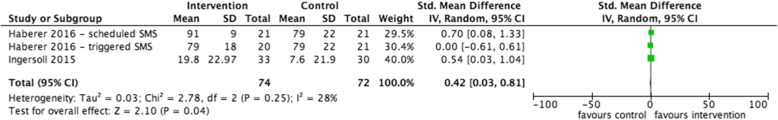
Adherence to HIV medication measured as MEM with interventions delivered by text message SMD

Figures 1 and 2 Forest plot of primary outcome adherence (measured as MEMs) in interventions delivered by text message. Relative risk and SMD.

### Interventions delivered by mobile phone call

Five trials evaluated interventions delivered by mobile phone call which reported a total of 12 outcomes [29, 38–41]. An improvement in adherence was measured in one of the 6 objective primary outcomes and in three of the 6 secondary subjective outcomes reported. One trial [38] showed a reduction in HIV viral load. The Huang [39] trial split the group into treatment naïve and treatment experienced. Two trials looked at participants that were not adherent [38, 41], only Belzer [38] improved adherence. No trials were similar enough to pool (Table 4).

**Table 4:** Summary of primary and secondary outcome of mobile phone interventions delivered by phone call

Frequency of Interventions delivered by phone call	Trial	Outcome	RR	SMD	LCI	UCI	*P* value
Primary outcome							
Weekly phone call	Uzma	HIV viral load	1.04		0.84	1.28	0.74
Weekly phone call	Uzma	Pill identification test	1.17		0.88	1.57	0.28
Daily phone-call	Belzer	HIV viral load difference		**−1.00**	**−1.89**	**−0.11**	**0.03**
Bi-weekly phone counseling	Kalichman	Pill count		6.30	−2.68	15.28	0.16
Fortnightly phone call	Huang naïve	CD4 count		10.00	−40.90	60.90	0.70
Fortnightly phone call	Huang experienced	CD4 count		−32.00	−103.19	39.19	0.39
Secondary outcome							
Fortnightly phone call	Kebaya	Adherence 6 weeks (questionnaire)	**1.26**		**1.07**	**1.47**	**0.00**
Weekly phone call	Uzma	Self reported adherence	1.11		0.90	1.37	0.33
Daily phone call	Belzer	Self-reported adherence 3 months	**4.86**		**1.22**	**19.28**	**0.03**
Daily phone-call	Belzer	Self-reported adherence		**47.74**	19.52	75.96	0.00
Fortnightly phone call	Huang naïve	Self-reported adherence		3.20	−2.14	8.54	0.22
Fortnightly phone call	Huang experienced	Self-reported adherence		0.10	−0.58	0.78	0.78

Clinically significant results (*P* < 0.05) have been highlighted in bold

Link to support was identified in 3 trials [38, 39, 41] and three or more BCTs were found in 3 trials [38, 40, 41]. Of these only Belzer [38] showed an improvement in adherence. Only Kalichman [41] explicitly stated a behaviour change model that underpinned the intervention. Mobile phone calls by nature were interactive.

### Interventions delivered by mobile phone imagery

One trial [42] reported four objective outcome results of interventions delivered by mobile phone imagery. One outcome showed a statistically significant improvement in HIV viral load (Table 5).

**Table 5:** Summary of primary outcome of mobile phone interventions delivered by images

Intervention	Trial	Primary outcome - continuous data	RR	SMD	LCI	UCI	*P* value
Daily imagery	Perera	Medication adherence		1.84	−0.40	4.08	0.06
Daily imagery	Perera	Prescribed doses taken		1.56	−1.99	5.12	0.32
Daily imagery	Perera	Pharmacy dispensing		6.80	.	.	.
Daily imagery	Perera	HIV viral load log 10		**−0.40**	**−0.78**	**−0.02**	**0.02**

Clinically significant results (*P* < 0.05) have been highlighted in bold

### Interventions delivered by mixed intervention

Four trials evaluated interventions delivered by mixed mechanism [43–46] and reported a total of 10 outcomes. There were six primary outcomes and four secondary outcomes. All of the outcomes except for the Shet trial [43] reported improvements in adherence. The Adbulrahman trial [46] reported a difference in mean adherence in the intervention group as statistically significant (*p* = 0.035). They also reported significant biological differences between the control and intervention group - a significantly higher rise in CD4 count (*p* = 0.017) in the intervention group and higher viral load in the control group (*p* = 0.001). In both Abdulrahman [46] and Maduka’s trial [45] we were unable to calculate RR or MD based on the data provided. Maduka’s report a statistically significant (95% CI *P* = 0.007) improvement in median CD4 count [45]. The Lester trial [44] which also involved text messaging and telephone follow up (for those requesting it or not responding) showed a statistically significant improvement in adherence (Table 6).

**Table 6:** Summary of primary and secondary outcome of mobile phone interventions delivered by mixed method

MIXED	Trial	Outcome	RR	SMD	LCI	UCI	*P* value
Primary							
Twice a week text message + monthly adherence counselling	Maduka	CD4 count change	**.**	.	.	.	.
Weekly voice message and pictoral	Shet	Virological failure	1.00		0.70	1.44	0.99
Weekly voice message and pictoral	Shet	Suboptimal adherence to ART (pill count)	1.24		0.93	1.65	0.14
Weekly text + counselling	Lester	Viral suppression	**1.18**		**1.01**	**1.40**	**0.04**
SMS and telephone call reminder	Abdulrahman	Viral load	**.**	.	.	.	.
SMS and telephone call reminder	Abdulrahman	CD4	**.**	.	.	.	.
Secondary							
Twice a week text message + monthly adherence counselling	Maduka	Self–reported adherence	**1.38**		**1.04**	**1.83**	**0.03**
Weekly text + counselling	Lester	ITT self-reported adherence > 95%	**1.24**		**1.06**	**1.44**	**0.01**
SMS and telephone call reminder	Abdulrahman	Self-reported adherence - good	**1.81**		**1.49**	**2.20**	**0.00**
SMS and telephone call reminder	Abdulrahman	Average adherence		**8.21**	**6.42**	**10.00**	**0.00**

Clinically significant results (*P* < 0.05) have been highlighted in bold

Interactivity was identified in three trials [43–45] of which two showed an effect. Link to support was identified in two trials [44, 45] all showed statistically significant improvements in adherence. Maduka [45] reported improvements in adherence in both objective and subjective measures (CD4 improvement and SRA). None of the mixed interventions used behaviour change models or reported having more than three or more BCTs as part of their interventions.

## Discussion

We identified 19 trials that investigated the effect of different mobile phone mechanisms on adherence to HIV medication. This review used a systematic approach, a replicable search strategy and standard systematic review methods [48] and is the first to include interventions delivered by mobile phone call. Previous reviews of mobile phone interventions designed to increase ART adherence have grouped all “mobile phone interventions that used any text messages” together without differentiating between interventions delivered via text message and mobile phone call, and BCTs used in the interventions were not described.

We present pooled analyses of objective outcomes and our review is the first to differentiate between objective and subjective adherence measures. Self-reported adherence outcomes may differentially overestimate benefits in the intervention group [49] due to lack of participant blinding, recall bias and the desire to please the provider [7]. We only pooled objective measures of the same outcome as such analyses allow the clinical benefits achieved for patients to be more clearly interpreted.

We found no effect when interventions delivered by text message were pooled in the RR, however, there was a moderate effect in SMD. There was substantial heterogeneity across the trials and individual trials reported objective improvements in adherence. It was unclear if the delivery mechanism (daily, weekly, scheduled or triggered mechanism in text messages) had an effect since, individually, the results were of mixed statistical significance. Text message interventions described as ‘interactive’ and using more than three BCTs all showed improvements in adherence. None of the trials had a low risk of bias.

Previous reviews have found that text messaging is effective in increasing adherence to ART [19, 22, 23], Finitsis et al. [21], reported a pooled OR of 1.48 (1.09 to 2.01) on any HIV outcome, however, objective and subjective outcomes were pooled across all types of intervention provided they included some text messaging. Although pooling in this way affords greater statistical power, use of subjective outcomes in trials where participants cannot be blinded may have resulted in over-estimated effects and it is difficult to identify which intervention components were effective. A similar methodology was used in Thakkar et al. [23] which concluded that mobile phone text message approximately doubles medication adherence in chronic disease. This review included trials [44, 45] combining text message with counselling, which may have inflated results [23]. Mayer et al. [22] also reported a larger SMD than the SMD we calculate in our review (SMD 0.87 vs. SMD 0.42), however, the authors included trials with a pre-post study design, converted all outcomes to SMD, and pooled all trials that included any text message.

We find the effect of text message-delivered daily prompts to take medicines to be inconclusive. This is consistent with the findings of other trials of text message-delivered daily prompts designed to increase adherence to oral contraception, TB medication, malaria prophylaxis or antibiotics, pooled RR 1.0 (CI 0.77–1.3) [31, 33, 50]. Intervention fatigue may explain the ineffectiveness of daily medication prompts.

All text message interventions with interactivity included in this review improved adherence, however we were unable to pool results (differing outcome measures and Hardy et al. [30] used an intervention as the control). This finding supports the conclusions of the Wald et al. [24] systematic review which explored the effects of two-way communication and interactivity in mobile phone-delivered interventions targeting adherence to any medication and concluded that interventions involving two-way text messaging improved medication adherence [24]. Mbuagbaw et al. also showed that interactivity improved adherence to ART [51] and Finnitis reports that interventions which include interactivity are more effective [21].

In our review, we distinguish between interactivity and a specific link to support from a person. These characteristics were heterogeneous, which is unsurprising given the nature of interactivity varied e.g. texting back to confirm you have taken medicine rather than texting back if you would like to speak to a health care provider, and the nature of the link to support could require passive or active involvement (a phone call from a health care provider because you requested one or because you didn’t respond or a telephone number to call if further advice was needed). Trials of interventions that involve sending a text message and providing phone follow up from a health care provider report increased uptake of long acting contraception and increase adherence to preventative medication for cardiovascular disease, as well as increase adherence to antiretroviral medication and reduce viral load [52, 53].

Among the five trials of interventions delivered by mobile phone call included in this review, only one reported a statistically significant reduction in viral load post intervention [32]. One trial using mobile phone imagery reported a reduction in HIV viral load. It is likely that the effect of interventions delivered by mobile phone call would be similar to the effect of adherence interventions delivered by landline - SMD in pooled behavioural outcomes 0.49 (− 1.12 to 2.11) I240%, [54]. The content of calls in both our review and the Cochrane review of phone calls was generally poorly described and is likely to be variable, resulting in different effects across trials [54]. In the one trial in our review which reported beneficial effects, the intervention was well described and involved confirming if medications were taken, providing problem-solving support, and referral to services to address adherence barriers if needed [38].

Of the mixed trials in our review, one trial delivered by mobile automated phone voice messaging showed no benefit, however, the other three mixed trials reported benefit, either in increasing CD4 count or reducing viral load [44–46]. All of the mixed interventions which included a link to support improved adherence, however, the time and costs involved requires clarification.

A wide range of other factors influence adherence to ART but have not been targeted in interventions to date. These factors include information about how medicines work, why they are important and how to take them, how to develop regular medicine taking habits, reassurance regarding common minor side effects and information about side effects for which help should be sought. The interventions in this review contained few BCTs (median 2 and maximum 6). In other areas such as smoking cessation effective behaviour change interventions delivered by text message included 19 BCTs [55].

Our review has some important limitations. With no existing gold standard objective measures of adherence [56, 57], trials included in this review used 15 different adherence measures limiting our ability to conduct pooled analyses of the same outcomes. There were also too few trials to conduct a meta-regression exploring all the factors which could influence heterogeneity of outcomes including: allocation concealment, blinding of outcome assessors, types of participant (treatment experienced/naive), factors influencing adherence targeted, BCT employed, mode of delivery, and duration of follow up.

Adherence measures may be at risk of the ‘Hawthorne effect’, where participants alter their behaviour due to awareness of being observed, especially if there is considerable contact in mid-trial follow-up points [58]. Self-reported measures and measures that can be manipulated in the short term such as pill count will be more susceptible to this effect.

It is also important to consider that in RCT’s the control group may have higher adherence levels by virtue of trial participation and increased surveillance, which may reduce the ability to detect true differences in the trial and thus underestimate intervention effects. In pragmatic trials there may be a trade-off between maintaining internal validity by achieving high follow up and achieving generalisability for “real world” purposes.

We coded the BCTs using an established taxonomy [25], however, coding was dependant on the authors’ description of the intervention, which often lacked detail. More comprehensive descriptions were requested but responses were limited, especially for transcripts of mobile phone call interventions. It is likely that content of mobile phone calls differed between trials which may influence the outcome of mobile phone counselling interventions.

Many of the trials had small sample sizes and were therefore underpowered to detect changes in the outcomes collected. As mentioned before, we did not pool analyses across different outcomes also resulting in reduced statistical power. The median follow-up time across trials was 4 months which is insufficient to determine the long-term impact of the intervention - some studies suggest that adherence slowly decreases with time due to pill fatigue [59].

WHOs current advice is there is high quality evidence for weekly text messages and they are effective to enhance adherence [60] . The evidence is more nuanced than this advice suggests, and new recommendations based on the updated evidence can now be made which recommend only specific interventions that have been shown to be effective.

Cost-analyses of existing effective interventions is needed prior to considering widespread implementation. Further clarification regarding the aims or content of phone calls would be helpful for services considering implementation. Future trials should include an exploration of the mechanism of action of interventions. The evidence base would be enhanced if a gold standard measure of ART adherence were agreed internationally. Interventions targeting a wider range of factors influencing adherence might have greater effects than existing interventions and should be evaluated by randomised controlled trial.

## Conclusions

Our review demonstrates text message improves adherence when measured as SMD but not RR. Interventions delivered by text message combined with health care provider mobile phone call have benefits on clinically important outcomes and text message interventions that include a link to a health care professional, interactivity and three or more BCTs all showed improvements in objective adherence measures. The evidence supports consideration of specific interventions shown to be effective for implementation, rather than mobile phone-based interventions in general. Interventions targeting a wider range of barriers to adherence and exploring other mechanisms may be more effective than existing interventions and may reduce the amount of health care provider input needed. Such interventions should be evaluated in a randomised controlled trial with long-term objective and clinically important outcomes alongside associated cost-effective analysis.

## Additional files

Additional file 1: PRISMA flow diagram of study selection (DOCX 42 kb)

Additional file 2: Search strategy for EMBASE (DOCX 15 kb)

Additional file 3: Abraham and Michie taxonomy of behaviour change technique (DOCX 108 kb)

Additional file 4: Summary of adherence measures used by each trial (DOCX 14 kb)

Additional file 5: Risk of bias comments (DOCX 41 kb)

Additional file 6: Funnel plot to show publication bias of the use of mobile phone interventions delivered by text message (DOCX 23 kb)

Additional file 7: Reasons for exclusion from the review (DOCX 21 kb)

## Abbreviations

ART: Anti-retroviral therapy
BCT: Behavioural change theory
MEM: Medication event monitoring
PLWH: People living with HIV
RCT: Randomised control trial
RR: Relative risk
SMD: Standardised mean difference
VAS: Visual analogue scale
WHO: World Health Organisation
