# Supplementary material for: A systematic review and meta-analysis in the effectiveness of mobile phone interventions used to improve adherence to antiretroviral therapy in HIV infection
Source: BMC Public Health. 2019 Jul 9;19:915. doi: 10.1186/s12889-019-6899-6 (PMC6617638; doi:10.1186/s12889-019-6899-6)
Supplement: Supplementary file 1 — PRISMA flow diagram of study selection (DOCX 42 kb) [file 12889_2019_6899_MOESM1_ESM.docx]

Additional file 1: PRISMA flow diagram of study selection

Studies included in quantitative synthesis (meta-analysis)
(n = 16 )

Studies included in qualitative synthesis
(n = 19 )

Full-text articles assessed for eligibility
(n = 46 )

Records excluded
(n = 212 )

Records screened
(n = 258 )

Records after duplicates removed
(n = 258 )

Additional records identified through other sources
(n = 9 )

## Identification

## Eligibility

## Included

## Screening

Records identified through database searching
(n = 511 )

Full-text articles excluded, with reasons
(n = 28 ) See Appendix 7
