# Supplementary material for: A systematic review and meta-analysis in the effectiveness of mobile phone interventions used to improve adherence to antiretroviral therapy in HIV infection
Source: BMC Public Health. 2019 Jul 9;19:915. doi: 10.1186/s12889-019-6899-6 (PMC6617638; doi:10.1186/s12889-019-6899-6)
Supplement: Supplementary file 2 — Search strategy for EMBASE (DOCX 15 kb) [file 12889_2019_6899_MOESM2_ESM.docx]

Additional file 2: Search strategy for EMBASE

1. (mobile adj2 phone$).tw.
2. (mobile adj2 telephone$).tw.
3. mobile application/
4. mobile phone/
5. wireless communication/
6. (application adj software).tw.
7. cell adj2 phone$.tw.
8. (cellular adj2 phone$).tw.
9. (cellular adj2 telephone$).tw.
10. ((blackberr$ or black-berr$) adj3 (mobile$ or phone$ or computer$)).tw.
11. (google adj3 phone$).tw.
12. (Text$ adj messag$).tw.
13. (short messag$ service$ or SMS).tw.
14. (MMS or multimedia messag$ service$).tw.
15. or /1-14
16. clinical trial/
17. randomized controlled trial/
18. randomization/
19. single blind procedure/
20. double blind procedure/
21. crossover procedure/
22. placebo/
23. randomi?ed controlled trial$.tw.
24. rct.tw.
25. Random allocation.tw.
26. Randomly allocated.tw.
27. Allocated randomly.tw.
28. (allocated adj2 random$).tw.
29. Single blind$.tw.
30. Double blind$.tw.
31. ((treble or triple) adj blind$).tw.
32. Placebo$.tw.
33. or/16-32
34. Case study/
35. Case report.tw.
36. Abstract report/ or letter/
37. Or/ 34-36
38. 33 not 37
39. exp human immunodeficiency virus/
40. Exp human immunodeficiency virus infection/
41. Hiv.tw.
42. human immunodeficiency virus.tw.
43. human immuno-deficiency virus. tw.
44. human immune-deficiency virus.tw.
45. acquired immune-deficiency syndrome.tw.
46. acquired immunedeficiency syndrome.tw.
47. acquired immuno-deficiency syndrome.tw.
48. AIDS.tw.
49. or/39-48
50. 15 AND 38 AND 49
