# Supplementary figures and images for: A systematic review and meta-analysis in the effectiveness of mobile phone interventions used to improve adherence to antiretroviral therapy in HIV infection
Source: BMC Public Health. 2019 Jul 9;19:915. doi: 10.1186/s12889-019-6899-6 (PMC6617638; doi:10.1186/s12889-019-6899-6)

Additional file 3: Abraham and Michie taxonomy of behaviour change technique.


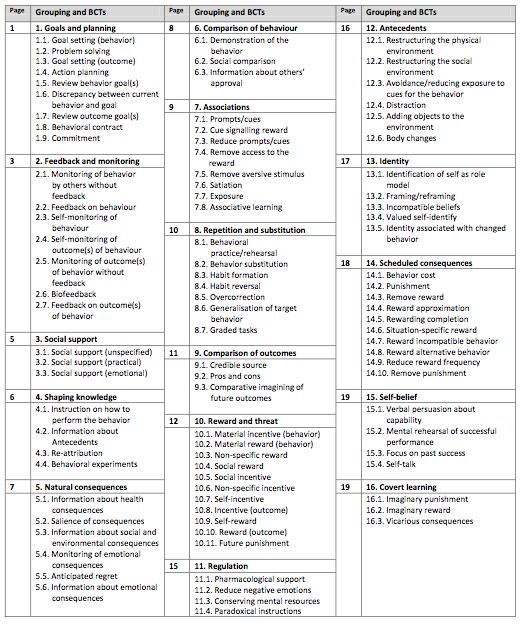

Supplement: Supplementary file 3 — Abraham and Michie taxonomy of behaviour change technique (DOCX 108 kb) [file 12889_2019_6899_MOESM3_ESM.docx]
