# Supplementary material for: A systematic review and meta-analysis in the effectiveness of mobile phone interventions used to improve adherence to antiretroviral therapy in HIV infection
Source: BMC Public Health. 2019 Jul 9;19:915. doi: 10.1186/s12889-019-6899-6 (PMC6617638; doi:10.1186/s12889-019-6899-6)
Supplement: Supplementary file 4 — Summary of adherence measures used by each trial (DOCX 14 kb) [file 12889_2019_6899_MOESM4_ESM.docx]

Additional file 4: Summary of adherence measures used by each trial.

| Measure | Trial |
| --- | --- |
| Self-reported adherence | Uzma, Maduka , Mbuagbaw, Lester, Hardy, Da Costa, Belzer, Kalichman , Huang, Kebaya, Nsagha, Orell, Abdulrahman |
| Sub-optimal adherence to ART | Shet |
| HIV viral load | Uzma, Perera , Belzer , Orrell, Abdulrahman |
| Viral suppression | Lester |
| Virological failure | Shet, Orrell |
| CD4 count | Maduka, Huang, Abdulrahman |
| Pill identification test | Uzma |
| MARS medication adherence | Perera |
| Prescribed doses taken | Perera |
| Pharmacy dispensing | Perera |
| VAS (Visual Analogue Scale) | Mbuagbaw |
| Pharmacy refill data | Mbuagbaw, Ingersoll |
| MEMs (Medication Event monitoring) adherence | Pop-eleches, Hardy, Da Costa, Sabin, Haberer, Orrell |
| Pill count | Hardy, Da Costa, Orrell |
| CAS (Composite adherence score) | Hardy |
