# Supplementary material for: A systematic review and meta-analysis in the effectiveness of mobile phone interventions used to improve adherence to antiretroviral therapy in HIV infection
Source: BMC Public Health. 2019 Jul 9;19:915. doi: 10.1186/s12889-019-6899-6 (PMC6617638; doi:10.1186/s12889-019-6899-6)
Supplement: Supplementary file 5 — Risk of bias comments (DOCX 41 kb) [file 12889_2019_6899_MOESM5_ESM.docx]

Additional file 5: Risk of bias comments

| **Trial** | **Randomization description** | **Allocation Concealment** | **Blinding description** | **Blinding primary outcomes – objective** | **Blinding secondary outcomes – subjective** | **Incomplete outcome description** | **Selective outcome reporting bias decision** | **Contamination description** | **Other - general comment** |
| --- | --- | --- | --- | --- | --- | --- | --- | --- | --- |
| Da Costa 2012 (31) | Randomly generated system automatically distributed patients into either control or intervention. | Not mentioned | The MDT who treated the patient and collected data on adherence only knew participant group allocation at the end of the trial. | Low risk of bias pill counting and MEM | High risk of bias self reported adherence | Only 21/29 remained in the trial (72.4%) | Outcome measures mentioned in the Methods are also in the results section. | Not mentioned | Trial only included those with cell phones, and literate patients |
| Pop-Eleches 2011 (32) | Randomization schedule prepared in advance by the investigator. A sequence of random number between 0 and 1 were generated. 4 equal intervals between 1 and 2/3 corresponded with the 4 intervention and values from 2/3 to 1 were control group. | Not mentioned | No mention of blinding but MEMS is electronic system so reduced bias | MEMs adherence, 48 hour interruption – low risk | No subjective measures – low risk | Consort diagram 92.8 low risk | Unclear | Not mentioned | High risk of bias - Randomization, allocation and blinding not discussed. Participants had to have a smart -phone, selection bias |
| Mbuagbaw 2012 (33) | Parallel group design, 1:1 Allocation ratio, computer generated randomization list, random block sizes of 2,4,6 | The allocation codes were then sequentially affixed to the phone numbers of consecutively recruited participants. This sequence was sent to the research centre by email and concealed in a password-protected computer until intervention was assigned. | Trained interviewers blinded to group allocations. Patients identified only by their phone number and sequential trial number. Data analysts were also blinded to the group allocation. | Low risk of bias – VAS, pharmacy refill data | Self reported – high risk of bias | Intention to treat, imputed techniques to handle missing data, loss to follow up 18% | Outcome measures mentioned in the Methods are also in the results section. | Not mentioned | Needed mobile phone to be part of trial, selection bias |
| Lester 2010 (44) | 1:1 simple randomization, random numbers generated by project statistician with a random number generating program | Sealed in individual opaque envelopes marked with trial identification | Laboratory assays and analyses were done by investigators masked to treatment allocation. | Viral suppression – low risk of bias | Self reported adherence - high risk of bias | Total attrition 19% , 23% , when mortality excluded 10 % and 11% LTFU so high risk of bias | Outcome measures mentioned in the Methods are also in the results section. | Several participants reported forwarding their weekly message to non-intervention to share support – high risk of bias | Loss to follow up calculations does not add up in the complete case analysis. |
| Hardy 2011 (30) | Team statistician who had no contact with trial subjects, a randomization list was created and given to the clinician who used it to assign each subject. | No mention of concealment | Combination of adherence measures used, unclear who did the pill count or MEMS | Low risk – MEMS, pill count | No subjective measures | Of the 23 subjects, 4 lost to follow up 17% | Outcome measures mentioned in the Methods are also in the results section. | Not mentioned | No mention of blinding, allocation concealment. Had to be English speaking to participate. Purposive sampling. |
| Sabin 2015 (34) | randomised within each stratum 1:1 , randomisation on site, block randomisation procedure where site coordinator pulled an unmarked allocation envelope | unmarked envelope | no blinding of participants | Adherence - Wisepill technologies number of opening signals. | CD4 cell and UDVL and change in CD4 cell. "All analysis conducted with knowledge of intervention assignment" | 3.3% loss to follow up | Low risk | no mention | 18+, owned a mobile phone, deemed at risk of poor adherence, $25 re-imbursement |
| Ingersoll 2015 (35) | random assignment generated in advance using a randomisation program. Randomised to either TEXT or usual care. | sealed envelope | not mentioned | Pharmacy refill data | Missed visits | 90.3% completed the 3 month post intervention follow-up | Low risk | No mention | given study phone so not to include text costs, had low adherence identified when they were screened over the phone or in private room. |
| Haberer 2016 (36) | simple random number generator 1:1:1 | no mention | no mention | Adherence - Wisepill technologies number of opening signals. | RNA suppression - blinding not mentioned | lost to follow up no contact or adherence data for 3 months. Missing adherence data - non adherent 94%. 6% lost to follow up. | Low risk | no mention | 18+ , Own personal cell phone with reliable reception, willing to identify 1 -2 social supporters, living less than 20km from MRRH |
| Nsagha 2016 (28) | purposive sampling. Serially numbered list of 90 participants, ballots prepared and randomly drew out numbers | no mention | no mention | No objective outcome reported | Self reported adherence | loss to follow up not reported – unclear | Low risk | no mention | 18+ able to read, owned a cellphone , |
| Orrell 2015 (37) | random number sequence was generated offsite | allocation to study arm was concealed in sealed individual opaque envelope which were numbered from 1 to 230 | envelope opened by study nurse, Blinded to the allocation, onsite | electronic pill Wisepill Technologies monitoring - objective blinding not mentioned | retention in care, virological failure and number of >72 hours treatment interruptions . Blinding not mentioned | lost to follow up if they did not attend clinic, had blood drawn or collected medication for more than 12 weeks. Also if they could not be traced. 8.3% LTFU and 3.5% died | Additionally reported self-report/ tablet return in the results section. | no mention | possession of own mobile phone, seen by clinic staff , compensation for their time |
| Belzer 2014 (38) | Randomised within sites, equal proportions using permuted block randomization. | Not mentioned | No explicit mention of blinding in this trial. | Viral load objective, low risk of bias. | Self reported adherence – high risk of bias | 7 in the intervention were discontinued from receiving calls out of 19 | Outcome measures mentioned in the Methods are also in the results section. | Not mentioned | Participants who missed more than 20% of calls a month in 2 consecutive months or who went off medication for 14 or more consecutive days were discontinued from the trial intervention. High risk of bias |
| Huang 2013 (39) | Low risk - permuted block of randomization in groups of 2 or 4 | Concealed in an opaque sealed envelope | Not mentioned explicitly but those who were allocated phone call intervention, received information about the intervention by a well-trained registered nurse/ health personnel who may have also been designated to deliver intervention. | CD4 count low risk | Self reported adherence – high risk | Follow up rate for treatment experienced 88.2% at 3 months | Unclear | Not mentioned | Selection bias - had to own mobile phone |
| Kebaya 2014 (29) | Not mentioned | Not mentioned | Not mentioned | Unclear | Unclear | Not mentioned | Not mentioned | Not mentioned |  |
| Uzma 2011 (40) | Random number table. A pencil was dropped on the page of a random number table with closed eyes and the number right beneath the tip of pencil was taken as the starting point. Low risk | Not mentioned | Not mentioned explicitly but the trial intervention delivered by the researcher and then self reported adherence was assessed by the researcher. | Pill identification test, HIV viral load, unclear if blinded | Self – reported adherence | High risk of bias 8/ 76 which is 10.5% | High risk of bias as CD4 count was not measured as intended as out of stock in the treatment centre. | Not mentioned | Needed PTCL or mobile phone to be in the trial. Exclusion criteria inability to give informed written consent |
| Kalichman 2011 (41) | Computer generated simple randomization scheme. | Not specified – unclear | Recruitment, screening and office based assessment staff remained blinded to condition. Adherence counsellor never conducted outcome assessments. The adherence counsellor used the adherence obtained from the pill-count as feedback for self-regulation counselling. | Low risk of bias, ART adherence unannounced pill counts | Medication adherence self efficacy  Unclear | 1 control lost to follow up, baseline characteristics initially did not show any significant difference, low risk of bias | Outcome measures mentioned in the Methods are also in the results section. | Unclear | Allocation concealment not mentioned. |
| Maduka 2013 (45) | random assignment using a randomisation function of the WINPEPI statistical software. | Study identification codes were sealed in individual opaque envelopes and placed in a bag. | Adherence counsellors and research assistants were blinded to the study group allocations. Self reported adherence high risk of bias, CD4 low. | CD4 low risk of bias | Self reported adherence – high risk of bias | Intention to treat analysis  low risk of bias  lost to follow up in total intervention 2 , control group 8.  10/ 104, so more than 90% in the study | Low risk of bias – purposive sampling to recruit participants at the clinic waiting area during the health education sessions | Study and intervention groups were assigned a separate clinic day throughout the course of the trial, so that study and intervention groups did not meet. | Selection bias - had to own a mobile phone |
| Shet 2014 (43) | 1:1 allocation ratio, stratified for sex permuted blocks of 4 or 6 | Sequentially numbered opaque sealed envelope | Researcher not involved in routine care measure pill count adherence outcome assessor blinded | Low risk of bias – virological failure, pill count | Low risk of bias | Loss to follow-up 7.6% intervention and 9.5% in control. Low risk of bias | Outcome measures mentioned in the Methods are also in the results section. | Low risk - Did not recruit people in the same household into the study to minimize contamination. | Selection bias as excluded those who expressed inability to attend study visits. |
| Abdulrahman 2017 (46) | simple complete based randomisation sequence technique in a 1:1 ratio. Independent bio-statistician. Random number program equal sets of unique numbers so group balance | sealed opaque enveloped | single blind parallel group RCT. The principal investigator and data analyst were blinded to the study arm | secondary outcomes - CD4 count, viral load, weight, TB status and OI index - no blinding mentioned | self reported adherence however considers number of missed drug doses and period of missed medication is less subjective. baseline measured in vitamin trial. AACTG specialised questionnaire | lost to follow up if did not show up for 3 consecutive months. Attendance had to be 5 out of 6. 93% completed 6 month follow-up assessment | Low risk | participants asked not to mention their group assignment or text messages to these staff - doesn't mention to other trial members | had to have telephone and be able to read text message. Only those who were adherent to the vitamin training were able to commence ART (selection bias) that meant that both groups had similar adherence, less likely that real life and control group measures would be inflated. |
| Perera 2014 (42) | Not mentioned | Not mentioned | Not mentioned | Viral load – low risk. Pharmacy dispensing | CAM – self reported so high risk | Consort diagram 92.8 low risk | Unclear | Not mentioned | High risk of bias - Randomization, allocation and blinding not discussed. Participants had to have a smart -phone, selection bias |
