# Supplementary material for: A systematic review and meta-analysis in the effectiveness of mobile phone interventions used to improve adherence to antiretroviral therapy in HIV infection
Source: BMC Public Health. 2019 Jul 9;19:915. doi: 10.1186/s12889-019-6899-6 (PMC6617638; doi:10.1186/s12889-019-6899-6)

Additional file 6: Funnel plot to show publication bias of the use of mobile phone interventions delivered by text message.


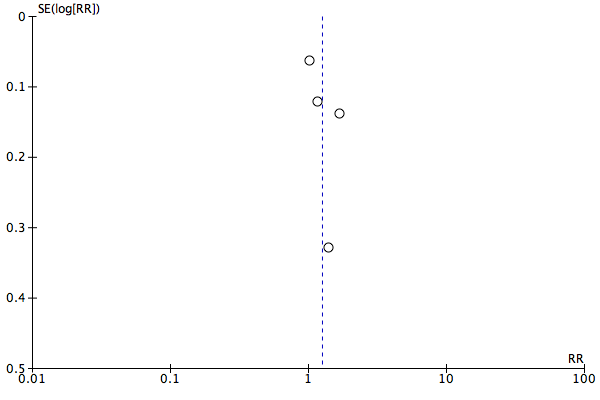

Supplement: Supplementary file 6 — Funnel plot to show publication bias of the use of mobile phone interventions delivered by text message (DOCX 23 kb) [file 12889_2019_6899_MOESM6_ESM.docx]
