# Supplementary material for: A systematic review and meta-analysis in the effectiveness of mobile phone interventions used to improve adherence to antiretroviral therapy in HIV infection
Source: BMC Public Health. 2019 Jul 9;19:915. doi: 10.1186/s12889-019-6899-6 (PMC6617638; doi:10.1186/s12889-019-6899-6)
Supplement: Supplementary file 7 — Reasons for exclusion from the review (DOCX 21 kb) [file 12889_2019_6899_MOESM7_ESM.docx]

Additional file 7: Reasons for exclusion from the review

| **Reference** | **Reason for exclusion** |
| --- | --- |
| Ammassari A, Trotta MP, Shalev N, Tettoni MC, Maschi S, et al. (2011) Timed short messaging service improves adherence and virological outcomes in HIV-1-infected patients with suboptimal adherence to antiretroviral therapy. J Acquir Immune Defic Syndr 58: e113–115. doi: 10.1097/qai.0b013e3182359d2a - | Not RCT, single arm trial |
| BELZER, M. 2013. A pilot study using cell phone interactions to improve HIV medication adherence in adolescents who have previously failed antiretroviral therapy. Journal of Adolescent Health, 1)**,** S7. | Abstract for presentation on the same study as the Belzer paper . |
| BELZER, M. E., MACDONELL, K. K., CLARK, L. F., HUANG, J., OLSON, J., KAHANA, S. Y., NAAR, S., SARR, M. & THORNTON, S. 2015. Acceptability and feasibility of a cell phone support intervention for youth living with HIV with nonadherence to antiretroviral therapy. AIDS Patient Care and STDs, 29**,** 338-345. | Feasibility study of the Belzer paper. |
| BIGNA, J. J., NOUBIAP, J. J., KOUANFACK, C., PLOTTEL, C. S. & KOULLA-SHIRO, S. Effect of mobile phone reminders on follow-up medical care of children exposed to or infected with HIV in Cameroon (MORE CARE): a multicentre, single-blind, factorial, randomised controlled trial. The Lancet Infectious Diseases, 14**,** 600-8. | This study looks at follow-up care and not adherence to ART |
| CHANG, L., KAGAAYI, J., AREM, H., NAKIGOZI, G., SSEMPIJJA, V., SERWADDA, D., QUINN, T., GRAY, R., BOLLINGER, R. & REYNOLDS, S. 2011. Impact of a mHealth Intervention for Peer Health Workers on AIDS Care in Rural Uganda: A Mixed Methods Evaluation of a Cluster-Randomized Trial. AIDS & Behavior, 15**,** 1776-1784. | Not about adherence to ART for HIV positive patients. |
| CHI, B. H. & STRINGER, J. S. 2010. Mobile phones to improve HIV treatment adherence. Lancet, 376**,** 1807-1808. | Article about trial |
| CHRISTOPOULOS, K. A., RILEY, E. D., TULSKY, J., CARRICO, A. W., MOSKOWITZ, J. T., WILSON, L., COFFIN, L. S., FALAHATI, V., AKERLEY, J. & HILTON, J. F. 2014. A text messaging intervention to improve retention in care and virologic suppression in a U.S. urban safety-net HIV clinic: study protocol for the Connect4Care (C4C) randomized controlled trial. BMC Infectious Diseases, 14. | Protocol |
| COLLIER, A. C., RIBAUDO, H., MUKHERJEE, A. L., FEINBERG, J., FISCHL, M. A. & CHESNEY, M. 2005. A randomized study of serial telephone call support to increase adherence and thereby improve virologic outcome in persons initiating antiretroviral therapy. Journal of Infectious Diseases, 192**,** 1398-1406. | Telephone intervention not mobile phone intervention |
| DICLEMENTE, R. J., WINGOOD, G. M., SALES, J. M., BROWN, J. L., ROSE, E. S., DAVIS, T. L., LANG, D. L., CALIENDO, A. & HARDIN, J. W. 2014. Efficacy of a telephone-delivered sexually transmitted infection/human immunodeficiency virus prevention maintenance intervention for adolescents: a randomized clinical trial. JAMA Pediatrics, 168**,** 938-946. | Telephone intervention not mobile phone intervention |
| DOWSHEN, N., KUHNS, L. M., GRAY, C., LEE, S. & GAROFALO, R. 2013. Feasibility of interactive text message response (ITR) as a novel, real-time measure of adherence to antiretroviral therapy for HIV+ youth. AIDS and behavior, 17**,** 2237-2243. | Feasibility study |
| DOWSHEN, N., KUHNS, L. M., JOHNSON, A., HOLOYDA, B. J. & GAROFALO, R. 2012. Improving adherence to antiretroviral therapy for youth living with HIV/AIDS: a pilot study using personalized, interactive, daily text message reminders. Journal of Medical Internet Research, 14**,** e51-e51. | Not randomised control trial |
| DRYDEN-PETERSON, S., BENNETT, K., HUGHES, M. D., VERES, A., JOHN, O., PRADHANANGA, R., BOYER, M., BROWN, C., SAKYI, B., VAN WIDENFELT, E., KEAPOLETSWE, K., MINE, M., MOYO, S., ASMELASH, A., SIEDNER, M., MMALANE, M., SHAPIRO, R. L. & LOCKMAN, S. 2015. An augmented SMS intervention to improve access to antenatal CD4 testing and ART initiation in HIV-infected pregnant women: A cluster randomized trial. PLoS ONE, 10. | Improve access to care not adherence to medication |
| FAIRLEY, C. K., LEVY, R., RAYNER, C. R., ALLARDICE, K., COSTELLO, K., THOMAS, C., MCARTHUR, C., KONG, D., MIJCH, A., MELBOURNE ADHERENCE, G. & MELBOURNE ADHERENCE, G. 2003. Randomized trial of an adherence programme for clients with HIV. Int J STD AIDS, 14**,** 805-9 | All participants in the intervention group did not receive a mobile phone intervention |
| Harris LT, Lehavot K, Huh D, Yard S, Andrasik MP, et al. (2010) Two-way text messaging for health behavior change among human immunodeficiency virus-positive individuals. Telemed J E Health 16: 1024–1029. doi: 10.1089/tmj.2010.0050 | Pager device not mobile phone |
| JONES, R., HOOUER, D. R. & LACROIX, L. J. 2013. A randomized controlled trial of soap opera videos streamed to smartphones to reduce risk of sexually transmitted human immunodeficiency virus (HIV) in young urban African American women. Nursing Outlook, 61**,** 205-215 | Participants were not HIV positive so to reduce risk rather than adherence |
| Lewis M, Uhrig J, Bann C, et al. Tailored Text Messaging Intervention for HIV Adherence: A Proof-of-Concept Study. Health Psychol. 2012 e-pup ahead of print. | Not randomised control trial |
| [David J. Moore](https://www.ncbi.nlm.nih.gov/pubmed/?term=Moore%20DJ%5BAuthor%5D&cauthor=true&cauthor_uid=25504449) et al. Individualized Texting for Adherence Building (iTAB): Improving Antiretroviral Dose Timing Among HIV-Infected Persons with Co-occurring Bipolar Disorder. AIDS Behav. 2015 Mar; 19(3): 459–471. | HIV and bipolar disease |
| PUCCIO, J. A., BELZER, M., OLSON, J., MARTINEZ, M., SALATA, C., TUCKER, D. & TANAKA, D. 2006. The use of cell phone reminder calls for assisting HIV-infected adolescents and young adults to adhere to highly active antiretroviral therapy: a pilot study. AIDS Patient Care & STDs, 20**,** 438-444. | Not randomised control trial |
| REYNOLDS, N. R., TESTA, M. A., SU, M., CHESNEY, M. A., NEIDIG, J. L., FRANK, I., SMITH, S., ICKOVICS, J., ROBBINS, G. K., GROUP, A. C. T. & TEAMS 2008. Telephone support to improve antiretroviral medication adherence: a multisite, randomized controlled trial. J Acquir Immune Defic Syndr, 47**,** 62-8 | Telephone not mobile phone intervention |
| Simoni JM, Huh D, Frick PA, Pearson CR, Andrasik MP, et al. (2009) Peer support and pager messaging to promote antiretroviral modifying therapy in Seattle: a randomized controlled trial. J Acquir Immune Defic Syndr 52: 465–473. doi: 10.1097/qai.0b013e3181b9300c - | Pager device not mobile phone |
| THIRUMURTHY, H. & T LESTER, R. 2012. M-health for health behaviour change in resource-limited settings: applications to HIV care and beyond. Bulletin of the World Health Organization, 90**,** 390-392. | Opinion article |
| Patricia Opondo Awiti, Alessandra Grotta, Mia van der Kop John Dusabe, Anna Thorson, Jonathan Mwangi Rino Belloco, Richard Lester Laura Ternent, Edwin Were and Anna Mia Ekström. The effect of an interactive weekly mobile phone messaging on retention  in prevention of mother to child transmission (PMTCT) of HIV program: study protocol for a randomized controlled trial (WELTEL PMTCT) BMC Medical Informatics and Decision Making (2016) 16:86 | Study protocol |
| Elena Salmoirago-Blotcher , Carla Rich , Rochelle K. Rosen, Shira Dunsiger, Aadia Rana , Michael P. Carey Phone-delivered mindfulness training to promote medication adherence and reduce sexual risk behavior among persons living with HIV: Design and methods. Contemporary Clinical Trials 53 (2017) 162–170 | Study protocol |
| Kate Jongbloed, Anton J. Friedman, Margo E. Pearce, Mia L. Van Der Kop, Vicky Thomas, Lou Demerai, Sherri Pooyak, Martin T. Schechter, Richard T. Lester, Patricia M. Spittal and The Cedar Project Partnership. The cedar project WelTel mHealth intervention for HIV prevention in young indigenous people who use illicit drugs”study protocol for a randomized controlled trial. Biomed Central (2016) 17:128 | Study protocol |
| Sheri A. Lippman, Starley B. Shade, Jeri Sumitani, Julia DeKadt, Jennifer M. Gilvydis, Mary Jane Ratlhagana, Jessica Grignon, John Tumbo, Hailey Gilmore, Emily Agnew, Parya Saberi, Scott Barnhart and Wayne T. Steward Evaluation of short message service and peer navigation to improve engagement in HIV care in South Africa: study protocol for a three-arm cluster randomized controlled trial. Biomed (2016) 17:68 | Study protocol |
| Elizabeth King, MD; Karen Kinvig, BN; Jonathan Steif, BSc; Annie Q Qiu, BSc; Evelyn J Maan, RN; Arianne YK Albert, PhD; Neora Pick, MD; Ariane Alimenti, MD; Mary H Kestler, MD; Deborah M Money, MD; Richard T Lester, MD; Melanie Caroline Margaret Murray, MD, PhD. Mobile Text Messaging to Improve Medication Adherence and Viral Load in a Vulnerable Canadian Population Living With Human Immunodeficiency Virus: A Repeated Measures Study J Med Internet Res 2017;19(6):e190) doi:10.2196/jmir.6631 | Not randomised control trial |
| Katerina A Christopoulos, Elise D Riley, Jacqueline Tulsky, Adam W Carrico, Judith T Moskowitz, Leslie Wilson, Lara S Coffin, Veesta Falahati, Jordan Akerley and Joan F Hilton A text messaging intervention to improve retention in care and virologic suppression in a U.S. urban safety-net HIV clinic: study protocol for the Connect4Care (C4C) randomized controlled trial BMC Infectious Diseases (2014) 14:718 | Study protocol |
| Nancy R. Reynolds, Veena Satyanarayana, Mona Duggal, Meiya Varghese, Lauren Liberti, Pushpendra Singh, Mohini Ranganathan, Sangchoon Jeon and Prabha S. Chandra. MAHILA: a protocol for evaluating a nurse- delivered mHealth intervention for women with HIV and psychosocial risk factors in India BMC Health Services Research (2016) 16:352 | Study protocol |
|  |  |
|  |  |
